# Lifespan and ROS levels in different *Drosophila melanogaster* strains after 24 h hypoxia exposure

**DOI:** 10.1242/bio.059386

**Published:** 2022-06-29

**Authors:** Sandro Malacrida, Federica De Lazzari, Simona Mrakic-Sposta, Alessandra Vezzoli, Mauro A. Zordan, Marco Bisaglia, Giulio Maria Menti, Nicola Meda, Giovanni Frighetto, Gerardo Bosco, Tomas Dal Cappello, Giacomo Strapazzon, Carlo Reggiani, Maristella Gussoni, Aram Megighian

**Affiliations:** 1 Institute of Mountain Emergency Medicine, Eurac Research, Via Ipazia 2, 39100 Bolzano, Italy; 2 MRC Mitochondrial Biology Unit, University of Cambridge, Cambridge Biomedical Campus, Cambridge CB2 0XY, UK; 3 Physiology, Genetics and Behaviour Unit, Department of Biology, University of Padova, 35131 Padova, Italy; 4 Institute of Clinical Physiology, National Research Council (CNR), 20162 Milan, Italy; 5 Department of Biomedical Sciences, University of Padova, via U. Bassi 58/B, 35131 Padova, Italy; 6 Department of Integrative Biology and Physiology, University of California, 610 Charles Young Drive East, Los Angeles, CA 90095-7239, USA; 7 Department of Biomedical Science, University of Padova, Via Marzolo 3, 35121 Padova, Italy; 8 Institute of Chemical Sciences and Technologies “G. Natta”-SCITEC, National Research Council, CNR-SCITEC, Via A. Corti 12, 20133 Milan, Italy; 9 Department of Biology, University of Padova, via U. Bassi 58/B, 35131 Padova, Italy; 10 Padova Neuroscience Center, University of Padova, via Orus 2/B, 35131 Padova, Italy

**Keywords:** *Drosophila melanogaster*, Wild-type strain, Hypoxia, Lifespan, ROS, EPR

## Abstract

During recent decades, model organisms such as *Drosophila melanogaster* have made it possible to study the effects of different environmental oxygen conditions on lifespan and oxidative stress. However, many studies have often yielded controversial results usually assigned to variations in *Drosophila* genetic background and differences in study design. In this study, we compared longevity and ROS levels in young, unmated males of three laboratory wild-type lines (Canton-S, Oregon-R and Berlin-K) and one mutant line (*Sod1^n1^*) as a positive control of redox imbalance, under both normoxic and hypoxic (2% oxygen for 24 h) conditions. Lifespan was used to detect the effects of hypoxic treatment and differences were analysed by means of Kaplan–Meier survival curves and log-rank tests. Electron paramagnetic resonance spectroscopy was used to measure ROS levels and analysis of variance was used to estimate the effects of hypoxic treatment and to assess ROS differences between strains. We observed that the genetic background is a relevant factor involved in *D. melanogaster* longevity and ROS levels. Indeed, as expected, in normoxia *Sod1^n1^* are the shortest-lived, while the wild-type strains, despite a longer lifespan, show some differences, with the Canton-S line displaying the lowest mortality rate. After hypoxic stress these variances are amplified, with Berlin-K flies showing the highest mortality rate and most evident reduction of lifespan. Moreover, our analysis highlighted differential effects of hypoxia on redox balance/unbalance. Canton-S flies had the lowest increase of ROS level compared to all the other strains, confirming it to be the less sensitive to hypoxic stress. *Sod1^n1^* flies displayed the highest ROS levels in normoxia and after hypoxia. These results should be used to further standardize future *Drosophila* research models designed to investigate genes and pathways that may be involved in lifespan and/or ROS, as well as comparative studies on specific mutant strains.

## Abstract (precis)

**Summary:** In our study *Drosophila melanogaster* was used to evaluate the effects of different environmental oxygen conditions on survival and ROS levels in three wild-type and one mutant strain.

## INTRODUCTION

Aerobic organisms require constant exposure to specific oxygen levels to maintain energy production and homeostasis. Since oxygen cannot be stored within most tissues, organisms are susceptible to events where oxygen supply is limited (hypoxia). Although oxygen deprivation highly affects general cellular homeostasis and, in the long term, may lead to cell death, most of the hypoxia-associated damages linked to short-term hypoxia have been shown to depend on the reoxygenation phase (Prag et al., 2020). The reestablishment of the normoxic environment leads to an increased and uncontrolled production of reactive oxygen species (ROS) (Granger and Kvietys, 2015; Chouchani et al., 2016). Impairments in the oxygen supply have been associated with different human pathologies, including heart and cerebral ischemia, pulmonary hypertension, obstructive sleep apnea, and high-altitude illnesses (such as high-altitude pulmonary edema and high-altitude cerebral edema) (Chen et al., 2020; Lavie, 2020). Therefore, understanding the molecular and physiological mechanisms of oxygen sensing and the correlated responses is important to develop targeted therapies.

Many studies have successfully shown that hypoxia impairs the redox state by increasing ROS levels, and concomitantly decreasing antioxidant capacity (Malacrida et al., 2019), but an accurate and detailed characterization of the underlying mechanisms is difficult to achieve in humans. Interesting insights into the pathophysiology of hypoxia can be obtained by studying simpler model organisms, such as *Drosophila melanogaster*. Using fruit flies as a model organism has many advantages, such as the short lifespan, simple reproductive cycle, and the high genetic manipulability. Moreover, *D. melanogaster* retains essential signaling pathways and cellular mechanisms of mammals, thus making *D. melanogaster* a suitable model to address biological questions relevant to human physiology and disease pathogenesis (Yamaguchi and Yoshida, 2018; Ugur et al., 2016), including the response to hypoxia and reoxygenation (Farahani and Haddad, 2003; Zhou et al., 2009; Zhou and Haddad, 2013).

Although adult fruit flies normally do not live at extremely low oxygen tensions, it has been shown that *D. melanogaster* effectively responds to variations in oxygen levels (Zhao and Haddad, 2011). However, differently from mammals, fruit flies can tolerate short hypoxic or even anoxic treatments without apparent injury (Zhao and Haddad, 2011; Azad and Haddad, 2009; Azad et al., 2009; Habib et al., 2021). Considering these features, the *D. melanogaster* organism is an ideal system to investigate the hypoxic response at its early stages, before the onset of major and irreversible damages to the organism. Canton-S (CS), Oregon-R (OR) and Berlin-K (BK) fly lines are considered classical, wild-type strains, which have often been used to study the cellular effects of aging, chronobiological, and behavioural aspects (Ganetzky and Flanagan, 1978; Helfrich-Förster, 2000; Grotewiel et al., 2005; Ruebenbauer et al., 2008). They all express a functional Sod1 and present a complete antioxidant defense and are usually used interchangeably. Inversely, the *Sod1^n1^* mutant line, carries a point mutation in the Sod1 protein leading to an unstable form of the cytosolic enzyme, which is rapidly degraded (Phillips et al., 1989, 1995). Sod1 is one of three Sod isoforms that act at cytoplasmatic level (Sod2 works in the mitochondrial matrix, while the Sod3 variant works in the extracellular milieu) as a central enzyme involved in the cellular redox balance, which detoxifies the superoxide anion, a highly reactive ROS species, by converting it into hydrogen peroxide (Wang et al., 2018). Lacking the enzymatic activity of Sod1, these mutants experience high ROS levels, premature mortality and are characterised by infertility and hypersensitivity to different oxidative insults (Phillips et al., 1989, 1995); thus, making them an excellent positive control of redox imbalance.

During the last few decades, different approaches have been developed to explore the hypoxic behavior of *D. melanogaster*. Some investigators have evaluated the effects of acute or chronic exposures to hypoxia (Azad et al., 2009; Zarndt et al., 2015; Polan et al., 2020; Sacks et al., 2018; Whelan et al., 2010; Feala et al., 2009; Habib et al., 2021), while other studies have assessed the impact of different patterns of hypoxia (i.e. intermittent hypoxia) (Azad et al., 2009), diet influence (Vigne and Frelin, 2006, 2007) or other environmental variables (Benasayag-Meszaros et al., 2015). However, differences in hypoxia generating experimental protocols (e.g. constant, or intermittent hypoxia) (Zhao and Haddad, 2011), in the experimental system used to generate the hypoxic condition, as well the large variety of fly strains (including many disease-associated fly models) has led to a difficult comparison of such studies. Recent advantages in *D. melanogaster* genetics and molecular biology have made it possible to compare and identify genes, pathways and differential regulation of gene expression that are known to affect hypoxic tolerance or susceptibility (Zhou and Haddad, 2013; Bacon et al., 1998).

This study was designed to investigate the effect of genetic background on ROS levels and fly lifespan in laboratory-controlled hypoxic conditions by tightly controlling for additional confounding factors (i.e. atmospheric pressure, temperature, humidity, light–dark regime etc.). We compared the longevity of three wild-type strains OR, CS, and BK and one mutant strain as a positive control of redox imbalance (*Sod1^n1^*) under normoxia and directly post-hypoxic exposure (2% oxygen for 24 h). The main aim was to determine whether classical, wild-type *D. melanogaster* strains can be used interchangeably when investigating phenotypes and/or genetic determinants of hypoxic exposure on longevity and ROS level, or whether specific genetic background effects could contribute to variation in response and difficulty in results replication.

## RESULTS

### Lifespan in normoxia conditions and after hypoxia exposure

**Fig. 1.**
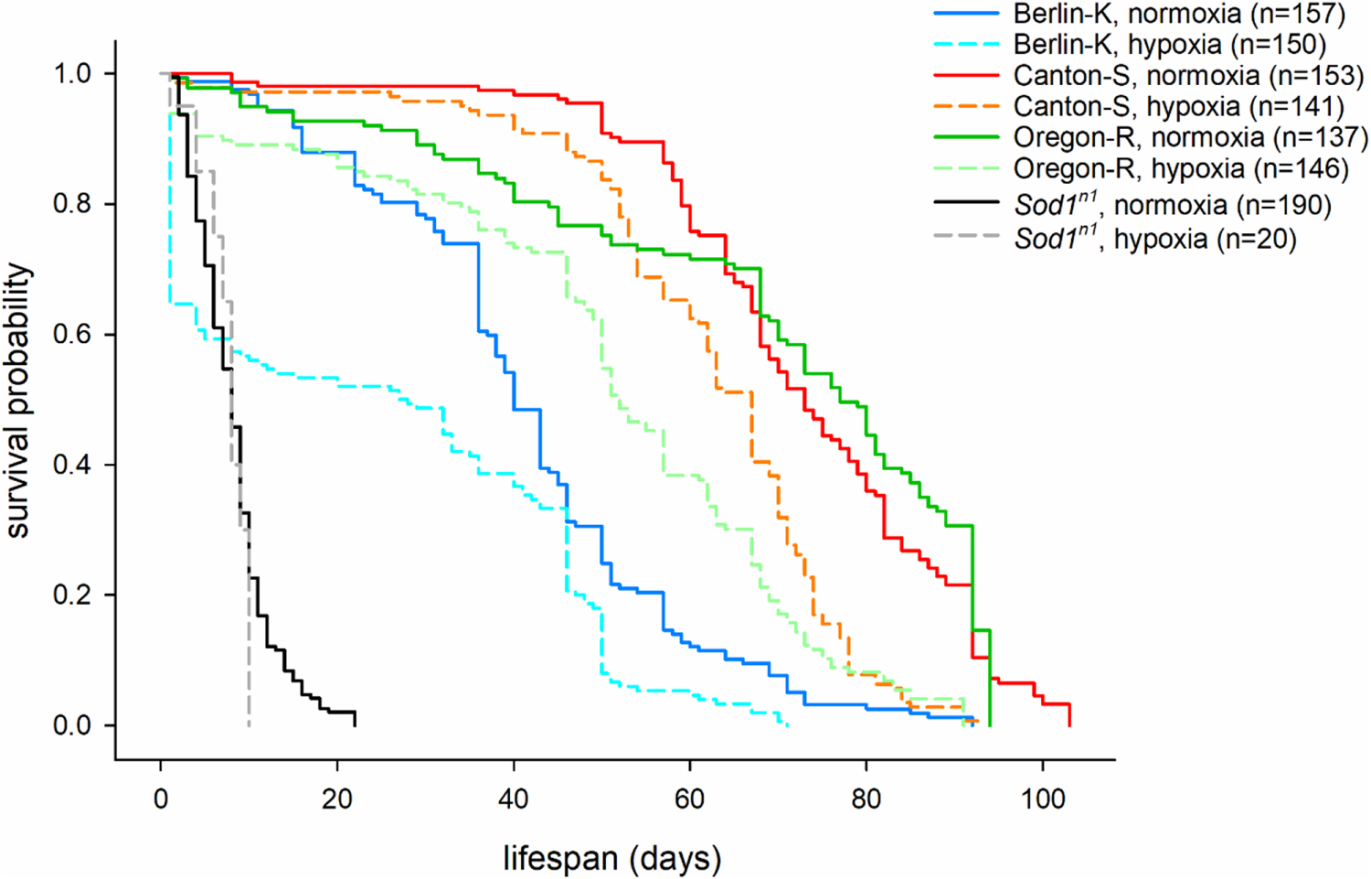
**Kaplan–Meier survival curves.** Survival curves for three wild-type strains (Berlin-K, Canton-S and Oregon-R) and the mutant *Sod1^n1^* under normoxia and hypoxia (2% of oxygen). For the experiments, only male adult flies were used (normoxia: Berlin-K *n*=157 flies; Canton-S *n*=153 flies; Oregon-R *n*=137 flies; *Sod1^n1^ n*=190 flies; hypoxia: Berlin-K *n*=150 flies; Canton-S *n*=141 flies; Oregon-R *n*=146 flies; *Sod1^n1^ n*=20 flies).

We observed that, under normoxia, lifespan differed both between wild-type strains (CS, OR, and BK) themselves, and in comparison, to the mutant strain (*Sod1^n1^*). Specifically, the BK fly line showed a shorter lifespan (*P*<0.001 for both comparisons, Fig. 1 and Table 1) and the higher mortality rate within 30 days (22%) compared with the OR and CS strains (11% and 2%, respectively) (*P*=0.074 and *P*<0.001, respectively; Table S1). OR and CS flies did not show a significant difference in lifespan (*P*=1; Table 1), although the latter showed a significant lower mortality rate within 30 days (*P*=0.015, Table S1) and somewhat a longer maximum lifespan (94 days versus 103 days) (Fig. 1). Inversely, the *Sod1^n1^* mutant displayed a significantly shorter lifespan (*P*<0.001; Fig. 1, and Table 1), and an extremely high mortality rate within 30 days (100%) compared to all the assessed wild-type strains (*P*<0.001; Fig. 1, and Table S1).

**Table 1.**
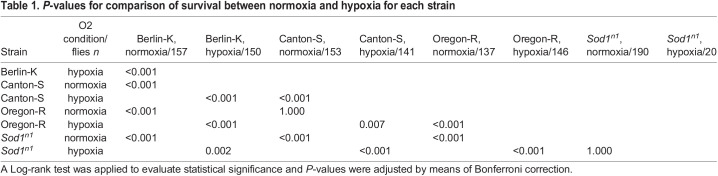
*P*-values for comparison of survival between normoxia and hypoxia for each strain

After assessing the longevity of each fly strain under normoxia, we further evaluated the impact of hypoxia and rapid reoxygenation. Lifespan of all the studied wild-type strains was significantly reduced after a 24 h exposure to 2% of oxygen atmosphere and reoxygenation (Fig. 1 and Table 1), though to a different degree. Mean survival time was 41 (95% CI 39-44) days in normoxia and 25 (95% CI 21-29) days in hypoxia for BK, 73 (95% CI 70-76) days in normoxia and 62 (95% CI 59-65) days in hypoxia for CS, and 69 (95% CI 65-73) days in normoxia and 50 (95% CI 47-54) days in hypoxia for OR. *Sod1^n1^* null mutant flies were not further affected by the hypoxic treatment [*P*=1; mean survival time 8 (95% CI 8-9) days in normoxia and 8 (95% CI 7-9) days in hypoxia]. The OR was more sensitive to hypoxia and reoxygenation compared to the CS line, resulting in a more evident reduction in lifespan (*P*=0.007) (Fig. 1, and Table 1) and a higher mortality rate within 30 days (18 versus 4%; *P*=0.001) (Table S1). BK strain appeared to be the most susceptible to the effects of hypoxia and reoxygenation when compared to both of the other wild-type lines (*P*<0.007) (Table 1), with the highest mortality rate within 1 day and 30 days after the treatment (35% and 51%) (Table S1). OR flies showed a similar mortality rate to *Sod1^n1^* mutants (6% versus 5%), while BK flies a higher mortality rate within 1 day than the *Sod1^n1^* mutants (35% versus 5%, *P*=0.027; Table S1 and Fig. 1).

### ROS in normoxia and after hypoxia exposure

**Fig. 2.**
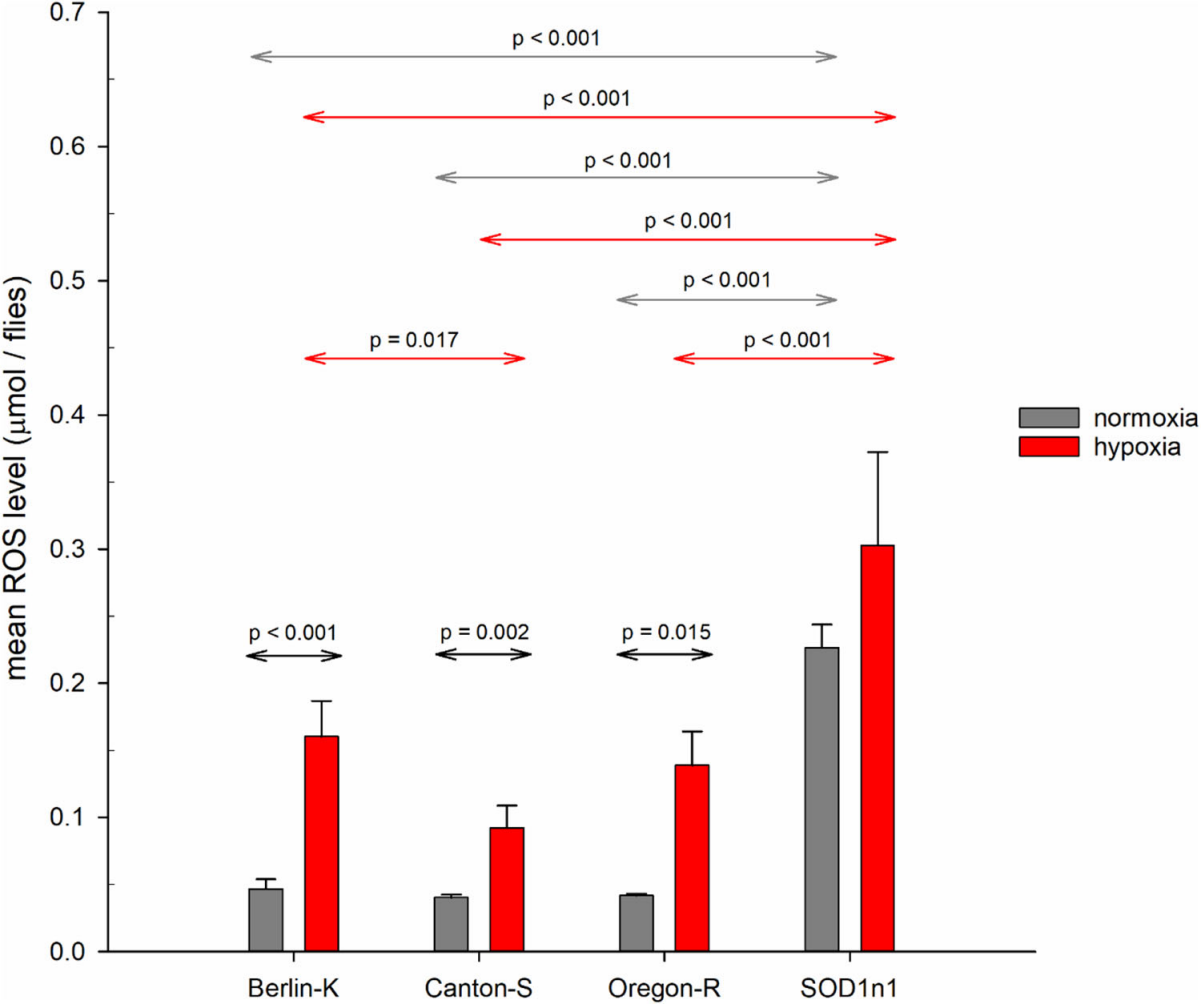
**Mean total body ROS levels measured using EPR.** ROS levels were measured in adult virgin males (4-5 days) for all the considered strains under normoxia [Berlin-K *n*=4 samples (total 100 flies); Canton-S *n*=3 (75 flies); Oregon-R *n*=2 (60 flies); *Sod1^n1^ n*=4 (100 flies)] and after 24 h of 2% of oxygen [Berlin-K *n*=7 samples (total 140 flies); Canton-S *n*=6 (120 flies); Oregon-R *n*=5 (100 flies); *Sod1^n1^ n*=4 (70 flies)]. Error bars represent standard deviation. For comparisons within normoxia and hypoxia *P*-values refer to *post hoc* tests of analysis of variance, while for comparisons between normoxia and hypoxia Student's *t*-test was used. *P*-values are adjusted by means of Bonferroni correction.

We quantified ROS levels in both flies maintained in normoxia and those exposed to hypoxia and rapid reoxygenation, both an effect of strain (*P*<0.001) and of O_2_ condition (*P*<0.001) on ROS were detected. We observed that under normoxic conditions all the analysed wild-type strains displayed a similar (low) basal level of ROS (*P*=1 for all three comparisons; Fig. 2 and Table S2), whereas *Sod1^n1^* mutant flies showed a significantly higher level of basal ROS compared to all the wild-type strains (*P*<0.001 for all three comparisons; Fig. 2 and Table S2).

After hypoxic treatment all strains demonstrated an increased ROS level, with BK flies having the highest increase (+243%), followed by OR (+232%) and CS (+129%), respectively (*P*<0.001 for BK, *P*=0.002 for CS and *P*=0.015 for OR; Fig. 2 and Table S2). In addition, the comparison of ROS between BK and CS line remained statistically significant after Bonferroni correction (*P*=0.017; Fig. 2 and Table S2). *Sod1^n1^* mutants had a significantly higher level of ROS compared to all the wild-type strains (*P*<0.001 for all three comparisons; Fig. 2 and Table S2) under each O_2_ conditions. However, the increase of ROS level (+34%) in the *Sod1^n1^* line after hypoxic treatment was not statistically significant (*P*=0.452; Fig. 2 and Table S2).

## DISCUSSION

In this study, we explored the effect of hypoxia (2% oxygen for 24 h) and rapid reoxygenation on lifespan and ROS level of young adult unmated males of different commonly used *Drosophila* wild-type strains (CS, OR and BK) and one mutant line (*Sod1^n1^*). We used male *Drosophila* for three reasons (i) their tissues are composed of postmitotic cells as are mammalian hearts and brains, (ii) their survival is independent of energy investment into egg production (Vigne and Frelin, 2006) (iii) their feeding behavior seems to be independent of the quality of the food (Min and Tatar, 2006). Moreover, we preferred to use unmated males because many studies highlight the importance of controlling mating status in any study with fruit fly because it can impact longevity, metabolism, and antioxidant status (Koliada et al., 2020). Finally, CS and OR lines were chosen as they usually serve as the wild-type background in which target mutations are studied (Iliadi et al., 2009), whereas the BK strain was extensively used in behavioral studies (Ruebenbauer et al., 2008). According to the literature and experience, the mutant strain (*Sod1^n1^*) was used as a positive control for longevity and ROS (Martin et al., 2009), and lifespan was specifically used as a discriminatory variable to study the effect of hypoxia and reoxygenation on different *Drosophila* lines. We found notable differences in longevity and ROS in whole body both between wild-type strains, as well as in contrast to the *Sod1^n1^* under both normoxic and hypoxic conditions. Moreover, our analysis highlighted differential effects of hypoxia reoxygenation on redox balance/unbalance. Together, these findings specifically indicate the potential for confounding results if different wild-type strains are employed in similar experimental protocols. Indeed, despite extensive funding attempts to better determine genetic background effects in *Drosophila* (https://app.dimensions.ai/details/grant/grant.2998107) and many publications trying to suggest optimal methodologies to uniform protocols (https://sites.google.com/a/umich.edu/pletcher-lab/protocols), it remains challenging to compare lifespan results reported in different papers. Too often data are obtained under different experimental conditions within different comparative *Drosophila* strains (i.e. sex, mating status, social status, dietary restriction, protocol for hypoxia and light–dark regime) (Habib, et al., 2021; Vigne and Frelin, 2006; Landis et al., 2020; Linford et al., 2013; Sun et al., 2013).

Although in this study we did not aim to explore the aging phenomenon directly, in contrast to previous papers (Ganetzky and Flanagan, 1978; Iliadi et al., 2009; Sanz et al., 2010), OR and CS male flies notably displayed a similar overall survivorship and mean survival time under normoxia, confirming that OR and CS are two long-lived strains (Bosco et al., 2015; Gubina et al., 2019). Conversely, the CS fly line presented a significantly lower mortality rate within 30 days, the period in which the flies are considered young, compared to all of the other strains. Furthermore, we observed that the BK line showed a significant shorter mean survival time and lifespan as compared to the OR and CS strains. As expected, *Sod1^n1^* mutant flies showed a significantly shorter mean survival time and lifespan, and a higher mortality rate within 30 days, compared to all of the assessed wild-type strains (Martin et al., 2009; Mockett et al., 2003; Reveillaud et al., 1994).

Despite the same origin, it is conceivable that our wild-type strains have a different genetic background compared to the strains used in other studies. All other conditions being equal, existing genetic background differences may indeed explain discrepancies among results obtained in different papers and, probably, the primary cause for such differences derives from founder effects rather than laboratory selection (Colomb and Brembs, 2014). All these findings support the idea that the genetic background is the most important characteristic regulating (affecting) the survivorship under normal oxygen conditions in *Drosophila* (Hunt et al., 2019; Aigaki et al., 2002).

A negative correlation between ROS levels and survivorship in flies has been previously reported (Sanz et al., 2010; Sohal et al., 1995; Arking, 2001), supporting one of the oldest, most widely discussed, modified and controversial theories (Harman, 1956; Orr et al., 2013; Lennicke and Cochemé, 2020; Shields et al., 2021) that an over-production of ROS can have an evident and strong contribution on aging in animals. Apparently in contrast with previously cited papers, our ROS analysis performed with the EPR method on the 3-4-day-old flies showed similar values under normoxia in all the three wild-type strains evaluated. Our data suggest that basal level of ROS alone does not negatively correlate with lifespan, according with other studies using mutant flies or protocols with a modified dietary regime (Miwa et al., 2004; Scialò et al., 2016; Owusu-Ansah et al., 2013) and the general idea that the relationship between ROS levels and lifespan is complex (Shields et al., 2021). *Sod1^n1^* mutant flies displayed the highest values of ROS and a drastic decrease in lifespan, which does not contradict the general idea that long-lived individuals or species should produce fewer ROS or better defense systems than short-lived ones (Sanz et al., 2010; Shields et al., 2021).

In the literature, there is a large collection of protocols and different assays to study hypoxia in *Drosophila* (Zhao and Haddad, 2011; Xia et al., 2018; Skandalis et al., 2011; Habib et al., 2021). However, in most of the reports, the impacts of environmental parameters on survival were neglected or not reported. Hence, we developed an efficient and reliable assay carefully controlling all environmental variables that could affect the comparability and reproducibility (including temperature, humidity and pressure) of our data, to assess the effect of hypoxia on survivorship and ROS level.

Our results are consistent with other published findings (Rascón and Harrison, 2010; Habib et al., 2021) and our previous experimental observations (Bosco et al., 2015), indicating that constant extreme hypoxia and rapid reoxygenation has detrimental effects on lifespan, independently of the *D. melanogaster* line considered. All of the wild-type strains studied suffered from a significant decrease in longevity, represented also by the reduction of the mean survival time, and the increase in mortality rate within 24 h and 30 days. However, we noticed that the magnitude of the negative effect due to the hypoxic stress and reoxygenation is strain specific. Indeed, the BK line showed the most evident reduction of lifespan among the wild-type strains. The OR strain seems to be more prone to the deleterious effects of hypoxia and reoxygenation affecting survivorship than the CS line, with the highest mortality rate within 24 h and 30 days. As expected, the mutant line *Sod1^n1^* was the most sensitive to the hypoxic stressor showing the shortest mean survival time, lifespan, and the highest mortality rate within 24 h and 30 days compared to OR and CS. Interestingly, the BK presented a dramatic increase in mortality rate during the first day compared not only to OR and CS but also to *Sod1^n1^*. In general, it was very difficult to obtain a comparable number of surviving flies in the *Sod1^n1^* line after the hypoxic test because most of them died during the experiment itself. The small number of surviving flies explains the failure to achieve a statistical significance for lifespan comparison after multiple test corrections. Variants in the genetic background of the BK line could be responsible for this extreme sensitivity to hypoxic and reoxygenation stress. Overall, the CS strain appears to be the best responder, with the highest recovery success and the longest lifespan after 24 h of extreme hypoxic stress. However, we cannot completely exclude the possible contribution of a differential regulation of gene expression or forms of epigenetics in response to such an extreme environmental stress.

Our results confirm that 24 h of extreme hypoxic stress increases ROS level (Rascón and Harrison, 2010; Habib et al., 2021). However, the increase in ROS level differs according to the strain considered. The BK line showed the highest rise followed by OR and CS lines. The CS strain displayed the lowest increase in ROS level compared to the other two wild-type strains, suggesting that CS flies may be less sensitive to hypoxic stress. In contrast, the mutant line *Sod1^n1^* had the absolute highest ROS level under normoxia, accompanied by the lowest increase after the hypoxic treatment, this result is probably affected by the low number of flies that survived after the hypoxic treatment and were sacrificed for the ROS level estimation. However, these findings highlight that the increase in ROS level due to oxygen deprivation seems to increase the potency of the negative effect of this environmental factor on *Drosophila* lifespan, gaining prominence with respect to other factors such as age, sex, and dietary regime (Habib et al., 2021; Deepashree et al., 2019). Moreover, all of these results confirm previous experimental observations demonstrating that oxidative stress generated by using dietary paraquat significantly affected longevity in different *D. melanogaster* strains (Vettraino et al., 2001; Vermeulen et al., 2005).

In conclusion, although we did not directly investigate the underlying buffering mechanisms, our findings suggest that the variation in genetic background appears to be the main factor limiting lifespan in *D. melanogaster* both under normoxic and hypoxic conditions. Moreover, the basal level of ROS measured in young unmated males of wild-type strains is not prognostic for lifespan duration. Nevertheless, with hypoxic exposure the contribution of ROS to a reduction in lifespan is further increased. Our results demonstrated that 24 h of extreme hypoxic exposure significantly reduces *Drosophila* lifespan with a different impact on the various wild-type strains tested. Moreover, in *Sod1^n1^* mutant flies, as expected, hypoxia exposure diminished the already short (with respect to wild-type flies) lifespan. These changes in *Drosophila* lifespan are concomitant with increased ROS levels, a finding that is particularly evident in *Sod1^n1^*-null mutant individuals.

These findings should be considered when attempting to further standardize future *Drosophila* research protocols designed to investigate genes and pathways that may be involved in lifespan and/or ROS level, as well as comparative studies on specific mutant strains. Our results suggest that the selection of a specific wild-type strain of *Drosophila* as a control can considerably affect the results and conclusions drawn from both aging and hypoxia studies; thus, going some way to explaining the contradictions that have frequently been found in the previous literature.

### Limits of the study

The current study has several caveats. ROS values in normoxia were estimated only at the initial stage (3-4 days post eclosion). Moreover, after the hypoxia treatment performed to test ROS levels, we were often unable to distinguish between dead flies and those that were only dormant, limiting the analysis for the post-hypoxic death rate. A low number of *Sod^n1^* flies were available for ROS level estimation after hypoxic treatment.

## MATERIAL AND METHODS

### *Drosophila* strains used in the study

In this study, we compared longevity and redox state in young, unmated males of three laboratory wild-type *D. melanogaster* lines (Canton-S, Oregon-R and Berlin-K) and one mutant line (*Sod1^n1^*) as a positive control of redox imbalance, under both normoxic and hypoxic (2% oxygen for 24 h) conditions.

As wild-type strains we selected CS, OR and BK fly lines that are considered classical, wild-type strains. According to original literature studies, OR stock derived from wild-type flies collected in 1925 by D. E. Lancefield at Roseburg (Oregon), while CS derived from wild flies collected in Canton (OH, USA) (Lindsley and Grell, 1968). The BK is a wild-type strain used extensively at Leiden University Medical Centre (Prof. J. Eeken and colleagues).

As a positive control of redox alteration, we exploited *Sod1^n1^* mutant flies, which carry a point mutation in the fly ortholog of the human superoxide dismutase enzyme (SOD1, referred to as Sod1 in *Drosophila*) at position 49, where a glycine is replaced by a serine. This substitution interferes with the process of dimerization, rendering the enzyme unstable and, therefore, inducing its rapid degradation (Phillips et al., 1995). Lacking the enzymatic activity of Sod1, these mutants experience high ROS levels, premature mortality and are hypersensitive to oxidative insults (Phillips et al., 1989). For all these reasons, *Sod1^n1^* mutant line was used to set the higher reading limit of the EPR instrument and was useful as a reference when measuring ROS levels in the different wild-type strains.

### *Drosophila* husbandry

The wild-type fruit fly strains OR (Professor A. Megighian laboratory stock, Department of Biology, University of Padova, Italy), CS (Professor A. Megighian laboratory stock), BK (kindly provided by Professor R. Wolf and Professor M. Heisenberg, Rudolf Virchow Center, University of Würzburg, Germany), and the mutant *Sod1^n1^* line (Bloomington *Drosophila* Stock Center, Indiana University, IN, USA) were reared on 12-15 ml of standard cornmeal medium in plastic vials (height: 12 cm; diameter, 2.5 cm) at 20±1°C and 60±10% relative humidity with a 12:12 h light: dark cycle. Every 3 days, the mating and egg laying vials, each containing 15 adult flies (ten males and five females) were emptied from the adults, which in turn were transferred to fresh ones, while the former vials, containing eggs but no adults, were kept for progeny collection. We selected adult virgin males daily, shortly after eclosion, and kept them in fresh vials at low density (∼10-15 flies) until testing. If the prompt selection of individuals was not possible, the vials were emptied before the next collection. The selected male flies were inspected daily to check their health and flipped to new vials every 2-3 days.

### Design of the hypoxia set-up

We developed an efficient and reliable assay keeping under control all environmental variables that could affect comparability and reproducibility (temperature, humidity, pressure, etc.) of our data, to assess the effect of hypoxia on survivorship and ROS levels in three wild-type strains (CS, OR and BK) and one mutant line (*Sod1^n1^*).

**Fig. 3.**
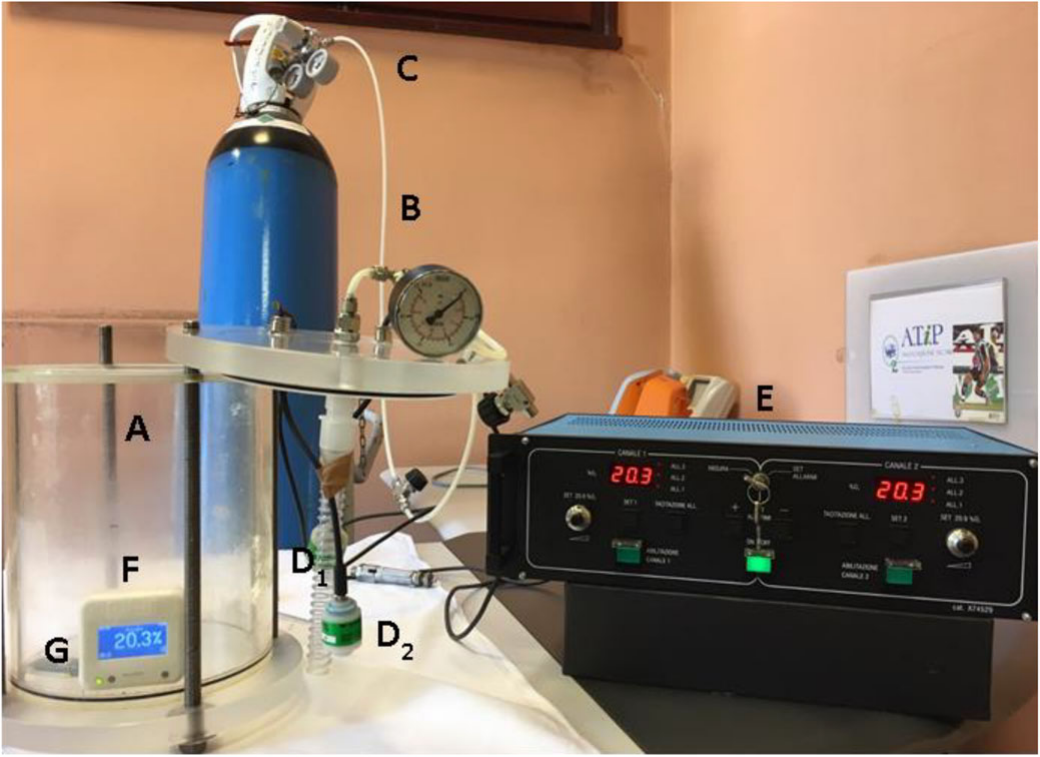
**Image illustrating the hypoxia-inducing chamber and gas apparatus.** A plexiglass cylinder (A) was used as a chamber to house the *Drosophila* during treatment. Nitrogen gas was introduced into the system by a tube (B) connected to a nitrogen tank (C). Two oxygen probes (D_1_ and D_2_) were connected to an external monitor (E). A third oxygen probe was in the CUBO_2_ device (F). Environmental conditions inside the chamber during experiments were monitored using a MSR145 data logger (G).

A self-constructed plexiglass cylinder was used as a chamber to house the flies during the hypoxia treatments (Fig. 3). To generate the hypoxic environment, nitrogen (N_2_) gas was injected into the chamber from the bottom of the chamber by a pipeline connected to an N_2_ tank. Three sensors were used to monitor the oxygen level inside the chamber. Two oxygen (O_2_) probes with a sensitivity range from 1% to 100% (R-17MED, Teledyne Analytical Instruments, CA, USA) were placed in the chamber and connected to an external monitor. A third oxygen probe, with a sensitivity range from 0% to 25%, was included in the CUBO_2_ device inside the chamber (Isolcell SpA, Bolzano, Italy). The environmental conditions (humidity, temperature, and pressure) inside the chamber were monitored by using an MSR^®^145 data logger (MSR Electronics GmbH, Switzerland).

### Experimental hypoxic protocol

To estimate the effects of the hypoxic treatment on *Drosophila* lifespan and ROS level, virgin male flies were collected, transferred into new vials containing standard fly food, and allowed to recover for 24 h before the hypoxic treatment. For the experiment, each vial containing 15 to 20 male flies was covered with gauze to allow gaseous exchanges to occur freely while stopping the flies from escaping. The vials were then transferred into the hypoxic chamber, which was flushed with a flow of pure nitrogen. On average it took 5 min to reach the final concentration of 2% oxygen inside the chamber. Flies were exposed to this oxygen concentration once for 24 h. All experiments were carried out in the same room, (Hyperbaric Medicine Center, Padova, Italy) maintaining the same levels of light and environmental noise. Room temperature and air temperature inside the chamber were the similar (20±1°C), and together with humidity (50-65%) were monitored throughout the experiments.

### Lifespan and mortality rate in normoxia and after hypoxia exposure

**Fig. 4.**
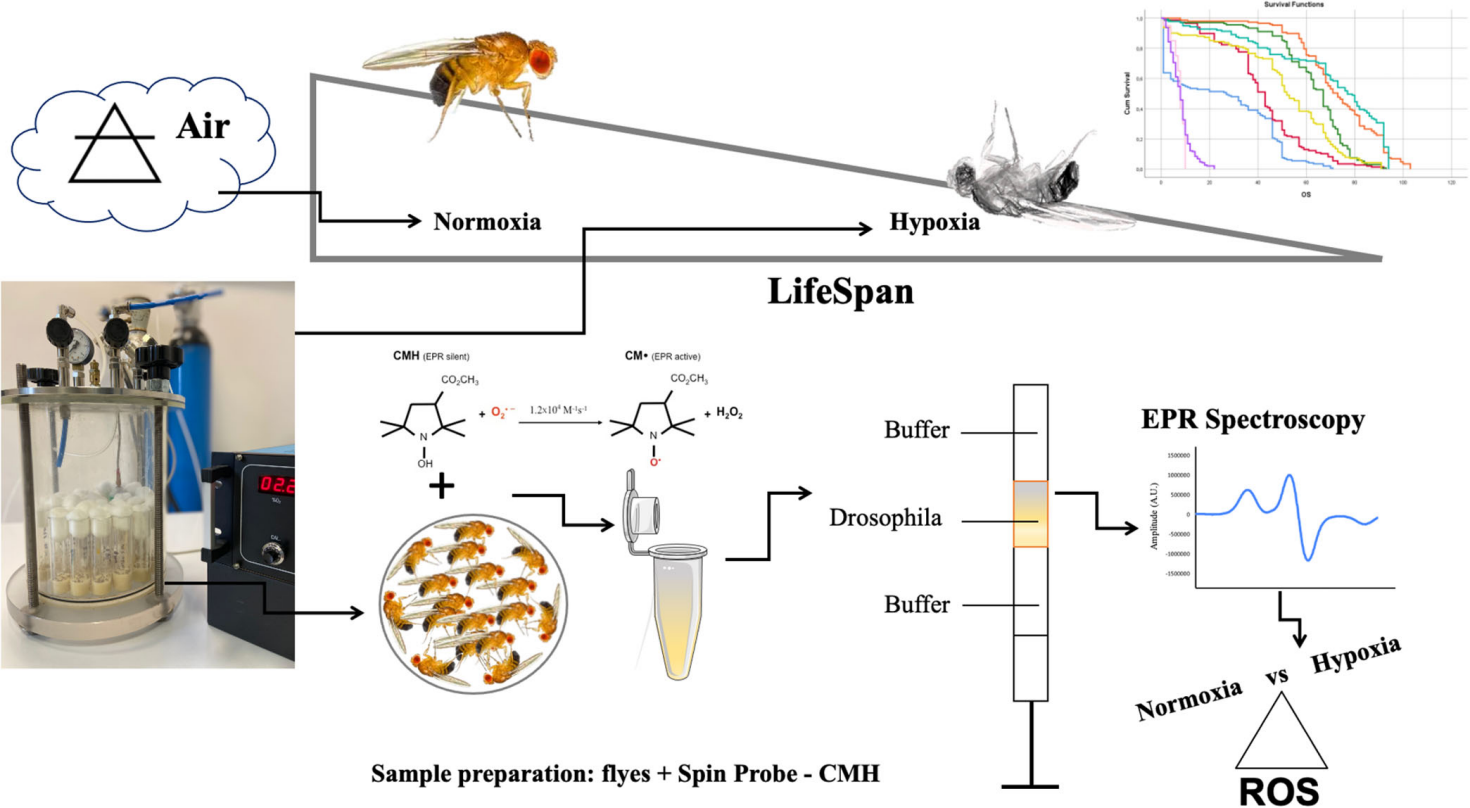
Outline of study design and experimental protocols used to collect and analyze *Drosophila* samples in the study.

To evaluate the effects of the hypoxic treatment on fly longevity, the lifespan of both hypoxia-treated and aged-matched untreated flies was analyzed by progressively counting the number of daily death events (Fig. 4). Briefly, for each strain, the number of dead flies was recorded daily, while surviving flies were transferred into new vials with fresh food every 2-3 days to avoid bacterial and/or mold growth. The procedure was repeated until there were no more living flies. Individuals that accidentally died or escaped during the transfer were not included in the analysis. Moreover, to better study the impact of hypoxia and reoxygenation on survival, the mortality rate was assessed within 1 day and after 30 days from hypoxia treatment (2% oxygen).

### ROS assay

EPR spectroscopy coupled with spin probes or traps enables quantitative determination of (ROS) and/or nitrogen species (RNS) (Gotham et al., 2020). Cyclic hydroxylamine spin probes react selectively with superoxide or other radicals to generate a nitroxide signal that can be quantified by EPR spectroscopy (Dikalov et al., 2018; Elajaili et al., 2019).

Based on this, we used EPR spectroscopy (Elajaili et al., 2019) technique to precisely quantify ROS at the tissue level (Berg et al., 2014); it is known in fact various exogenous factors like temperature, pressure, stress, can produce oxidative stress within the body and hence generates ROS (Nayak and Mihra, 2019). Various exogenous factors like temperature, sound, pressure, microbe infection and chemicals can produce oxidative stress within the body. We specifically measured the superoxide anion, which is considered a primary form of ROS abundantly produced upon reoxygenation, with cyclic hydroxylamine spin probes. In this manner, we obtained a snapshot of this specific ROS for each fly strain after exposure to hypoxia as compared to the normoxic condition.

### EPR spectroscopy for ROS assessment

Being a small animal, to quantify ROS in whole body of *Drosophila m.*, we used an X-Band (∼9.8 GHz) EPR spectrometer (e-Scan Bruker,BioSpin, GmbH, MA, USA). Hypoxic-treated, and untreated flies were anesthetized by cooling on ice (4°C) for 1 min (Constantinou et al., 2016), rapidly homogenized with a pellet pestle (Kontes; 749521-1500) on ice, blended with solution contained: Krebs-Hepes buffer (KHB) containing 25 μM deferoxamine methane-sulfonate salt (DF) chelating agent and 5 μM sodium diethyldithio-carbamate trihydrate (DETC) at pH 7.4 with 1 mM 1-hydroxy-3-methoxycarbonyl-2,2,5,5-tetramethylpyrrolidine (CMH, Noxygen Science Transfer and Diagnostics, Germany) as a spin-probe which is able to trap superoxide anions, and immediately incubated in a thermostatic water (37°C). After exactly 30 min of incubations, the time necessary to rich plateau, the samples were placed in the center of 1 ml plastic syringe, according to methods previously reported (Dikalov et al., 2018; Rivellini et al., 2021), snap frozen and stored at −80°C. The frozen block was removed by gentle push from the warmed-up syringe and analyzed in the quartz dewar with liquid nitrogen. Spectra were recorded at 77K. The EPR signal is generated by the reaction of the spin probe (CMH) with whole-body ROS. The spectrometer acquisition parameters used were the following: modulation amplitude, 9.08 G; centered field, 2.0023 ***g***; sweep time, 10.49 s; field sweep, 60 G; microwave power, 43.69 mW; number of scans, 40; receiver gain, 3.17×10^3^. All data were, in turn, converted into absolute concentration levels (micromoles) by adopting CP• (3-carboxy-2,2,5,5-tetramethyl-1-pyrrolidinyloxy) stable radical as external reference.

Data were normalized to the total number of flies present in the sample. All EPR spectra by sample flies were acquired within 48 h of euthanization. The acquired spectra were analyzed by using Win EPR software supplied by spectrometer manufacturer (Fig. 4).

Limits of detection (LOD) and limits of quantification (LOQ) were estimated using the ICH Guidelines [ICH Harmonised Tripartite Guideline, Validation on Analytical Procedures: Text and Methodology, Q2(R1), Step 4, 2005], that defines these parameters as the analytic concentrations at which the signal-noise ratios (SNR) are at least 3:1 and 10:1. Using the EPR method, LOD and LOQ depend upon the acquisition parameters, especially on the number of scans (NS), that influences linearly the SNR and the experimental time (Mrakic-Sposta et al., 2012). In the EPR spectrum with ROS at known concentration (6 μM) and recorded under the same acquisition parameters adopted in the present study, the SNR of the line belonging to the ROS signal with NS=40 was found to be 240. Therefore, LOD and LOQ are calculated as 6 μM×3/240=7.5×10^−2^ μM, and 6 μM×10/240=25×10^−2 ^μM, respectively.

### Statistical analysis

Lifespan differences were analysed by means of Kaplan–Meier survival curves and log-rank test. Survival times are reported as mean (95% confidence interval, CI). To study the effects of environmental stressors mortality rates within 1 day and 30 days were analyzed (Grotewiel et al., 2005; Bonilla et al., 2002). The mortality rates of the four strains were compared by means of Pearson's chi-squared test and Fisher's exact test was used for pairwise comparisons. A two-way analysis of variance (ANOVA) was used to detect effects of hypoxic treatment and strain on ROS. One-way ANOVA was performed to assess ROS differences between strains in each oxygen condition (normoxia and after hypoxia exposure) using *post hoc* tests for pairwise comparisons. Independent samples Student's *t*-test was performed to detect ROS differences between normoxia and hypoxia for each strain. *P*-values were adjusted by means of Bonferroni correction. SPSS version 26 (IBM Corporation, Armonk, NY, USA) was used for the statistical analysis, and *P<*0.05 (two-sided) was considered statistically significant.

## Supplementary Material

Supplementary information

## References

[BIO059386C1] Aigaki, T., Seong, K.-H. and Matsuo, T. (2002). Longevity determination genes in Drosophila melanogaster. *Mech. Ageing Dev.* 123, 1531-1541. 10.1016/S0047-6374(02)00089-1 12470891

[BIO059386C2] Arking, R. (2001). Gene expression and regulation in the extended longevity phenotypes of Drosophila. *Ann N. Y. Acad. Sci.* 928, 157-167. 10.1111/j.1749-6632.2001.tb05645.x 11795506

[BIO059386C3] Azad, P. and Haddad, G. G. (2009). Survival in acute and severe low O_2_ environment: use of a genetic model system. *Ann. N. Y. Acad. Sci.* 1177, 39-47. 10.1111/j.1749-6632.2009.05045.x 19845605

[BIO059386C4] Azad, P., Zhou, D., Russo, E. and Haddad, G. G. (2009). Distinct mechanisms underlying tolerance to intermittent and constant hypoxia in Drosophila melanogaster. *PLoS ONE* 4, e5371. 10.1371/journal.pone.0005371 19401761 PMC2670512

[BIO059386C5] Bacon, N. C. M., Wappner, P., O'Rourke, J. F., Bartlett, S. M., Shilo, B., Pugh, C. W. and Ratcliffe, P. J. (1998). Regulation of the Drosophila bHLH-PAS protein Sima by hypoxia: functional evidence for homology with mammalian HIF-1 alpha. *Biochem. Biophys. Res. Commun.* 249, 811-816. 10.1006/bbrc.1998.9234 9731218

[BIO059386C6] Benasayag-Meszaros, R., Risley, M. G., Hernandez, P., Fendrich, M. and Dawson-Scully, K. (2015). Pushing the limit: examining factors that affect anoxia tolerance in a single genotype of adult D. melanogaster. *Sci. Rep.* 5, 9204. 10.1038/srep09204 25777190 PMC4361850

[BIO059386C7] Berg, K., Ericsson, M., Lindgren, M. and Gustafsson, H. (2014). A high precision method for quantitative measurements of reactive oxygen species in frozen biopsies. *PLoS ONE* 9, e90964. 10.1371/journal.pone.0090964 24603936 PMC3947958

[BIO059386C8] Bonilla, E., Medina-Leendertz, S. and Díaz, S. (2002). Extension of life span and stress resistance of Drosophila melanogaster by long-term supplementation with melatonin. *Exp. Gerontol.* 37, 629-638. 10.1016/S0531-5565(01)00229-7 11909680

[BIO059386C9] Bosco, G., Clamer, M., Messulam, E., Dare, C., Yang, Z., Zordan, M., Reggiani, C., Hu, Q. and Megighian, A. (2015). Effects of oxygen concentration and pressure on Drosophila melanogaster: oxidative stress, mitochondrial activity, and survivorship. *Arch. Insect. Biochem. Physiol.* 88, 222-234. 10.1002/arch.21217 25529352

[BIO059386C10] Chen, P.-S., Chiu, W.-T., Hsu, P.-L., Lin, S.-C., Peng, I.-C., Wang, C.-Y. and Tsai, S.-J. (2020). Pathophysiological implications of hypoxia in human diseases. *J. Biomed. Sci.* 27, 63. 10.1186/s12929-020-00658-7 32389123 PMC7212687

[BIO059386C11] Chouchani, E. T., Pell, V. R., James, A. M., Work, L. M., Saeb-Parsy, K., Frezza, C., Krieg, T. and Murphy, M. P. (2016). A unifying mechanism for mitochondrial superoxide production during ischemia-reperfusion injury. *Cell Metab.* 23, 254-263. 10.1016/j.cmet.2015.12.009 26777689

[BIO059386C12] Colomb, J. and Brembs, B. (2014). Sub-strains of Drosophila Canton-S differ markedly in their locomotor behavior. *F1000Res* 3, 176. 10.12688/f1000research.4263.1 25210619 PMC4156027

[BIO059386C13] Constantinou, C., Apidianakis, Y., Psychogios, N., Righi, V., Mindrinos, M. N., Khan, N., Swartz, H., Szeto, H. H., Tompkins, R. G., Rahme, L. et al. (2016). In vivo high-resolution magic angle spinning magnetic and electron paramagnetic resonance spectroscopic analysis of mitochondria-targeted peptide in Drosophila melanogaster with trauma-induced thoracic injury. *Int. J. Mol. Med.* 37, 299-308. 10.3892/ijmm.2015.2426 26648055 PMC4716799

[BIO059386C14] Deepashree, S., Niveditha, S., Shivanandappa, T. and Ramesh, S. R. (2019). Oxidative stress resistance as a factor in aging: evidence from an extended longevity phenotype of Drosophila melanogaster. *Biogerontology* 20, 497-513. 10.1007/s10522-019-09812-7 31054025

[BIO059386C15] Dikalov, S. I. ; Polienko, Y. F. and Kirilyuk, I. (2018). Electron paramagnetic resonance measurements of reactive oxygen species by cyclic hydroxylamine spin probes. *Antioxid. Redox Signal.* 28, 1433-1443. 10.1089/ars.2017.7396 29037084 PMC5910043

[BIO059386C16] Elajaili, H. B., Hernandez-Lagunas, L., Ranguelova, K., Dikalov, S. and Nozik-Grayck, E. (2019). Use of electron paramagnetic resonance in biological samples at ambient temperature and 77 K. *J. Vis. Exp.* 11, e58461. 10.3791/58461 PMC8015406 30688300

[BIO059386C17] Farahani, R. and Haddad, G. G. (2003). Understanding the molecular responses to hypoxia using Drosophila as a genetic model. *Respir. Physiol. Neurobiol.* 135, 221-229. 10.1016/S1569-9048(03)00049-1 12809621

[BIO059386C18] Feala, J. D., Coquin, L., Zhou, D., Haddad, G. G., Paternostro, G. and McCulloch, A. D. (2009). Metabolism as means for hypoxia adaptation: metabolic profiling and flux balance analysis. *BMC Syst. Biol.* 9, 91. 10.1186/1752-0509-3-91 PMC2749811 19740440

[BIO059386C19] Ganetzky, B. and Flanagan, J. R. (1978). On the relationship between senescence and age-related changes in two wild-type strains of Drosophila melanogaster. *Exp. Gerontol.* 13, 189-196. 10.1016/0531-5565(78)90012-8 99324

[BIO059386C20] Gotham, J. P., Li, R., Tipple, T. E., Lancaster, J. R., Jr, Liu, T. and Li, Q. (2020). Quantitation of spin probe-detectable oxidants in cells using electron paramagnetic resonance spectroscopy: to probe or to trap? *Free Radic. Biol. Med.* 154, 84-94. 10.1016/j.freeradbiomed.2020.04.020 32376456 PMC7368495

[BIO059386C21] Granger, D. N. and Kvietys, P. R. (2015). Reperfusion injury and reactive oxygen species: the evolution of a concept. *Redox. Biol.* 6, 524-551. 10.1016/j.redox.2015.08.020 26484802 PMC4625011

[BIO059386C22] Grotewiel, M. S., Martin, I., Bhandari, P. and Cook-Wiens, E. (2005). Functional senescence in Drosophila melanogaster. *Ageing Res. Rev.* 4, 372-397. 10.1016/j.arr.2005.04.001 16024299

[BIO059386C23] Gubina, N., Naudi, A., Stefanatos, R., Jove, M., Scialo, F., Fernandez-Ayala, D. J., Rantapero, T., Yurkevych, I., Portero-Otin, M., Nykter, M. et al. (2019). Essential physiological differences characterize short- and long-lived strains of Drosophila melanogaster. *J. Gerontol. A. Biol. Sci. Med. Sci.* 74, 1835-1843. 10.1093/gerona/gly143 29945183

[BIO059386C24] Habib, P., Jung, J., Wilms, G. M., Kokott-Vuong, A., Habib, S., Schulz, J. B. and Voigt, A. (2021). Posthypoxic behavioral impairment and mortality of Drosophila melanogaster are associated with high temperatures, enhanced predeath activity and oxidative stress. *Exp. Mol. Med.* 53, 264-280. 10.1038/s12276-021-00565-3 33564101 PMC8080651

[BIO059386C25] Harman, D. (1956). Aging: a theory based on free radical and radiation chemistry. *J. Gerontol.* 11, 298-300. 10.1093/geronj/11.3.298 13332224

[BIO059386C26] Helfrich-Förster, C. (2000). Differential control of morning and evening components in the activity rhythm of Drosophila melanogaster--sex-specific differences suggest a different quality of activity. *J. Biol. Rhythms.* 15, 135-154. 10.1177/074873040001500208 10762032

[BIO059386C27] Hunt, L. C., Jiao, J., Wang, Y. D., Finkelstein, D., Rao, D., Curley, M., Robles-Murguia, M., Shirinifard, A., Pagala, V. R., Peng, J. et al. (2019). Circadian gene variants and the skeletal muscle circadian clock contribute to the evolutionary divergence in longevity across Drosophila populations. *Genome Res.* 29, 1262-1276. 10.1101/gr.246884.118 31249065 PMC6673717

[BIO059386C28] Iliadi, K. G., Iliadi, N. N. and Boulianne, G. L. (2009). Regulation of Drosophila life-span: effect of genetic background, sex, mating and social status. *Exp. Gerontol.* 44, 546-553. 10.1016/j.exger.2009.05.008 19481597

[BIO059386C29] Koliada, A., Gavrilyuk, K., Burdylyuk, N., Strilbytska, O., Storey, K. B., Kuharskii, V., Lushchak, O. and Vaiserman, A. (2020). Mating status affects Drosophila lifespan, metabolism and antioxidant system. *Comp. Biochem. Physiol. A Mol. Integr. Physiol.* 246, 110716. 10.1016/j.cbpa.2020.110716 32339661

[BIO059386C30] Landis, G. N., Doherty, D. and Tower, J. (2020). Analysis of Drosophila melanogaster Lifespan. *Methods Mol. Biol.* 2144, 47-56. 10.1007/978-1-0716-0592-9_4 32410023 PMC8016145

[BIO059386C31] Lavie, L. (2020). Intermittent Hypoxia and Obstructive Sleep Apnea: Mechanisms, Interindividual Responses and Clinical Insights. Chapter 3. In *Atherosclerosis, Arteriosclerosis and Arteriolosclerosis* (ed. L. Gianturco), pp. 1-12. London: IntechOpen Limited. https://www.intechopen.com/chapters/66924. 10.5772/intechopen.86117

[BIO059386C32] Lennicke, C. and Cochemé, H. M. (2020). Redox signalling and ageing: insights from Drosophila. *Biochem. Soc. Trans*. 48:367-377. 10.1042/BST20190052 32196546 PMC7200633

[BIO059386C33] Lindsley, D. L. and Grell, E. H. (1968). *The Mutants of Drosophila melanogaster. Genetic Variations of Drosophila melanogaster*. Washington, DC: Publications of the Carnegie Institution. 627, 469. http://publicationsonline.carnegiescience.edu/publications_online/genetic_variations.pdf.

[BIO059386C34] Linford, N. J., Bilgir, C., Ro, J. and Pletcher, S. D. (2013). Measurement of lifespan in Drosophila melanogaster. *J. Vis. Exp.* 7, 50068. 10.3791/50068 PMC3582515 23328955

[BIO059386C35] Malacrida, S., Giannella, A., Ceolotto, G., Reggiani, C., Vezzoli, A., Mrakic-Sposta, S., Moretti, S., Turner, R., Falla, M., Brugger, H. et al. (2019). Transcription factors regulation in human peripheral white blood cells during hypobaric hypoxia exposure: an *in-vivo* experimental study. *Sci. Rep.* 9, 9901. 10.1038/s41598-019-46391-6 31289332 PMC6617471

[BIO059386C36] Martin, I., Jones, M. A. and Grotewiel, M. (2009). Manipulation of Sod1 expression ubiquitously, but not in the nervous system or muscle, impacts age-related parameters in Drosophila. *FEBS Lett.* 583, 2308-2314. 10.1016/j.febslet.2009.06.023 19540235 PMC2719956

[BIO059386C37] Min, K.-J. and Tatar, M. (2006). Drosophila diet restriction in practice: do flies consume fewer nutrients? *Mech. Ageing Dev.* 127, 93-96. 10.1016/j.mad.2005.09.004 16256171

[BIO059386C38] Miwa, S., Riyahi, K., Partridge, L. and Brand, M. D. (2004). Lack of correlation between mitochondrial reactive oxygen species production and life span in Drosophila. *Ann. N. Y. Acad. Sci.* 1019, 388-391. 10.1196/annals.1297.069 15247051

[BIO059386C39] Mockett, R. J., Radyuk, S. N., Benes, J. J., Orr, W. C. ; and Sohal, R. S. (2003). Phenotypic effects of familial amyotrophic lateral sclerosis mutant Sod alleles in transgenic Drosophila. *Proc. Natl. Acad. Sci. USA* 100, 301-306. 10.1073/pnas.0136976100 12502789 PMC140958

[BIO059386C40] Mrakic-Sposta, S., Gussoni, M., Montorsi, M., Porcelli, S. and Vezzoli, A. (2012). Assessment of a standardized ROS production profile in humans by electron paramagnetic resonance. *Oxid. Med. Cell. Longev.*, 2012, 973927. 10.1155/2012/973927 22900129 PMC3412105

[BIO059386C41] Nayak, N. and Mishra, M. (2019). Estimation of oxidative stress and survorship in Drosophila Cap 11. In *Fundamental Approaches to Screen Abnormalities in Drosophila, Springer Protocols Handbooks* (ed. M. Mishra), pp. 123-134. New York: Springer. 10.1007/978-1-4939-9756-5_11

[BIO059386C42] Orr, W. C., Radyuk, S. N. and Sohal, R. S. (2013). Involvement of redox state in the aging of Drosophila melanogaster. *Antioxid Redox Signal.* 19, 788-803. 10.1089/ars.2012.5002 23458359 PMC3749695

[BIO059386C43] Owusu-Ansah, E., Song, W. and Perrimon, N. (2013). Muscle mitohormesis promotes longevity via systemic repression of insulin signaling. *Cell* 155, 699-712. 10.1016/j.cell.2013.09.021 24243023 PMC3856681

[BIO059386C44] Phillips, J. P. ; Campbell, S. D., Michaud, D., Charbonneau, M. and Hilliker, A. J. (1989). Null mutation of copper/zinc superoxide dismutase in Drosophila confers hypersensitivity to paraquat and reduced longevity. *Proc. Natl. Acad. Sci. USA* 86, 2761-2765. 10.1073/pnas.86.8.2761 2539600 PMC286998

[BIO059386C45] Phillips, J. P., Tainer, J. A., Getzoff, E. D., Boulianne, G. L., Kirby, K. and Hilliker, A. J. (1995). Subunit-destabilizing mutations in Drosophila copper/zinc superoxide dismutase: neuropathology and a model of dimer disequilibrium. *Proc. Natl. Acad. Sci. USA* 92, 8574-8578. 10.1073/pnas.92.19.8574 7567977 PMC41008

[BIO059386C46] Polan, D. M., Alansari, M., Lee, B. and Grewal, S. S. (2020). Early-life hypoxia alters adult physiology and reduces stress resistance and lifespan in Drosophila. *J. Exp. Biol.* 223, jeb226027. 10.1242/jeb.226027 32988998 PMC10668336

[BIO059386C47] Prag, H. A., Kula-Alwar, D., Beach, T. E., Gruszczyk, A. V., Burger, N. and Murphy, M. P. (2020). Mitochondrial ROS production during ischemia-reperfusion injury Chapter 26. In *Oxidative Stress* (ed. Helmut Sies), pp. 513-538. Amsterdam: Academic press, Elsevier. ISBN 9780128186060. 10.1016/B978-0-12-818606-0.00026-2

[BIO059386C48] Rascón, B. and Harrison, J. F. (2010). Lifespan and oxidative stress show a non-linear response to atmospheric oxygen in Drosophila. *J. Exp. Biol.* 213, 3441-3448. 10.1242/jeb.044867 20889824

[BIO059386C49] Reveillaud, I., Phillips, J., Duyf, B., Hilliker, A., Kongpachith, A. and Fleming, J. E. (1994). Phenotypic rescue by a bovine transgene in a Cu/Zn superoxide dismutase-null mutant of Drosophila melanogaster. *Mol. Cell. Biol.* 14, 1302-1307. 10.1128/mcb.14.2.1302-1307.1994 8289809 PMC358485

[BIO059386C50] Rivellini, C., Porrello, E., Dina, G., Mrakic-Sposta, S., Vezzoli, A., Bacigaluppi, M., Gullotta, G. S., Chaabane, L., Leocani, L., Marenna, S. et al. (2021). JAB1 deletion in oligodendrocytes causes senescence-induced inflammation and neurodegeneration in mice. *J. Clin. Invest.* 7, 145071. 10.1172/JCI145071 PMC8803330 34874913

[BIO059386C51] Ruebenbauer, A., Schlyter, F., Hansson, B. S., Löfstedt, C. and Larsson, M. C. (2008). Genetic variability and robustness of host odor preference in Drosophila melanogaster. *Curr. Biol.* 18, 1438-1443. 10.1016/j.cub.2008.08.062 18804372

[BIO059386C52] Sacks, D., Baxter, B., Campbell, B. C. V., Carpenter, J. S., Cognard, C., Dippel, D., Eesa, M., Fischer, U., Hausegger, K., Hirsch, J. A. et al. (2018). Multi Society Consensus Quality Improvement Revised Consensus Statement for Endovascular Therapy of Acute Ischemic Stroke. *Int. J. Stroke* 13, 612-632. 10.1177/1747493018778713 29786478 10.1177/1747493018778713

[BIO059386C53] Sanz, A., Fernández-Ayala, D. J. M., Stefanatos, R. K. A. and Jacobs, H. T. (2010). Mitochondrial ROS production correlates with, but does not directly regulate lifespan in Drosophila. *Aging* 2, 200-223. 10.18632/aging.100137 20453260 PMC2880708

[BIO059386C54] Scialò, F., Sriram, A., Fernández-Ayala, D., Gubina, N., Lõhmus, M., Nelson, G., Logan, A., Cooper, H. M., Navas, P., Enríquez, J. A. et al. (2016). Mitochondrial ROS produced via reverse electron transport extend animal lifespan. *Cell. Metab.* 23, 725-734. 10.1016/j.cmet.2016.03.009 27076081 PMC4835580

[BIO059386C55] Shields, H. J., Traa, A. and Van Raamsdonk, J. M. (2021). Beneficial and detrimental effects of reactive oxygen species on lifespan: a comprehensive review of comparative and experimental studies. *Front. Cell. Dev. Biol.* 9, 628157. 10.3389/fcell.2021.628157 33644065 PMC7905231

[BIO059386C56] Skandalis, D. A., Stuart, J. A. and Tattersall, G. J. (2011). Responses of Drosophila melanogaster to atypical oxygen atmospheres. *J. Insect. Physiol.* 57, 444-451. 10.1016/j.jinsphys.2011.01.005 21241703

[BIO059386C57] Sohal, R. S., Sohal, B. H. and Orr, W. C. (1995). Mitochondrial superoxide and hydrogen peroxide generation, protein oxidative damage, and longevity in different species of flies. *Free Radic. Biol. Med.* 19, 499-504. 10.1016/0891-5849(95)00037-X 7590400

[BIO059386C58] Sun, Y., Yolitz, J., Wang, C., Spangler, E., Zhan, M. and Zou, S. (2013). Aging studies in Drosophila melanogaster. *Methods Mol. Biol.* 1048, 77-93. 10.1007/978-1-62703-556-9_7 23929099 PMC4664065

[BIO059386C59] Ugur, B., Chen, K. and Bellen, H. J. (2016). Drosophila tools and assays for the study of human diseases. *Dis Model. Mech.* 9, 235-244. 10.1242/dmm.023762 26935102 PMC4833332

[BIO059386C60] Vermeulen, C. J., Van De Zande, L. and Bijlsma, R. (2005). Resistance to oxidative stress induced by paraquat correlates well with both decreased and increased lifespan in Drosophila melanogaster. *Biogerontology* 6, 387-395. 10.1007/s10522-005-4903-2 16518700

[BIO059386C61] Vettraino, J., Buck, S. and Arking, R. (2001). Direct selection for paraquat resistance in Drosophila results in a different extended longevity phenotype. *J. Gerontol. A. Biol. Sci. Med. Sci.* 56, 415-425. 10.1093/gerona/56.10.B415 11584026

[BIO059386C62] Vigne, P. and Frelin, C. A. (2006). Low protein diet increases the hypoxic tolerance in Drosophila*.* *PLoS ONE* 1, e56. 10.1371/journal.pone.0000056 17183686 PMC1762395

[BIO059386C63] Vigne, P. and Frelin, C. (2007). Diet dependent longevity and hypoxic tolerance of adult Drosophila melanogaster. *Mech. Ageing Dev.* 128, 401-406. 10.1016/j.mad.2007.05.008 17606290

[BIO059386C64] Wang, Y., Branicky, R., Noë, A. and Hekimi, S. (2018). Superoxide dismutases: Dual roles in controlling ROS damage and regulating ROS signaling. *J. Cell. Biol.* 217, 1915-1928. 10.1083/jcb.201708007 29669742 PMC5987716

[BIO059386C65] Whelan, J., Burke, B., Rice, A., Tong, M. and Kuebler, D. (2010). Sensitivity to seizure-like activity in Drosophila following acute hypoxia and hypercapnia. *Brain Res.* 1316, 120-128. 10.1016/j.brainres.2009.12.036 20034480

[BIO059386C66] Xia, Y., Xu, W., Meng, S., Lim, N. K. H., Wang, W. and Huang, F.-D. (2018). An efficient and reliable assay for investigating the effects of hypoxia/anoxia on Drosophila. *Neurosci. Bull.* 34, 397-402. 10.1007/s12264-017-0173-7 28866769 PMC5856710

[BIO059386C67] Yamaguchi, M. and Yoshida, H. (2018). Drosophila as a model organism. *Adv. Exp. Med. Biol.* 1076, 1-10. 10.1007/978-981-13-0529-0_1 29951811

[BIO059386C68] Zarndt, R., Piloto, S., Powell, F. L., Haddad, G. G., Bodmer, R., Ocorr, K. (2015). Cardiac responses to hypoxia and reoxygenation in Drosophila. *Am. J. Physiol. Regul. Integr. Comp. Physiol.* 309, 1347-1357. 10.1152/ajpregu.00164.2015 PMC4698404 26377557

[BIO059386C69] Zhao, H. W. and Haddad, G. G. (2011). Review: Hypoxic and oxidative stress resistance in Drosophila melanogaster. *Placenta* 32 Suppl. 2, 104-108. 10.1016/j.placenta.2010.11.017 PMC3073591 21353099

[BIO059386C71] Zhou, D. and Haddad, G. G. (2013). Genetic analysis of hypoxia tolerance and susceptibility in Drosophila and humans. *Annu. Rev. Genomics Hum. Genet.* 14, 25-43. 10.1146/annurev-genom-091212-153439 23808366 PMC12990993

[BIO059386C72] Zhou, D., Visk, D. W. and Haddad, G. G. (2009). Drosophila, a golden bug, for the dissection of the genetic basis of tolerance and susceptibility to hypoxia. *Pediatr. Res.* 66, 239-247. 10.1203/PDR.0b013e3181b27275 19542900 PMC6620046

