# Supplementary material for: Lifespan and ROS levels in different Drosophila melanogaster strains after 24 h hypoxia exposure
Source: Biol Open. 2022 Jun 29;11(6):bio059386. doi: 10.1242/bio.059386 (PMC9253781; doi:10.1242/bio.059386)
Supplement: Supplementary information [file biolopen-11-059386-s1.pdf]

Table S1. Comparison of mortality rate within 1 day and 30 days of four different *Drosophila* strains in normoxia and hypoxia (Pearson's chi-squared test). *P*-values of pairwise comparisons (Fisher's exact test) are adjusted by means of Bonferroni correction.

Days Mortality Rate	O_2 condition	Berlin-K	Canton-S	Oregon-R	*$Sod1^{n1}$*	*p*-value Pearson's chi-squared test	Berlin-K *vs.* Canton-S	Berlin-K *vs.* Oregon-R	Berlin-K *vs.* *$Sod1^{n1}$*	Canton-S *vs.* Oregon-R	Canton-S *vs.* *$Sod1^{n1}$*	Oregon-R *vs.* *$Sod1^{n1}$*
1	Normoxia	1%	0%	1%	1%	0.795	-	-	-	-	-	-
	Hypoxia	35%	1%	6%	5%	**<0.001**	**<0.001**	**<0.001**	**0.027**	0.365	1.000	1.000
30	Normoxia	22%	2%	11%	100%	**<0.001**	**<0.001**	0.074	**<0.001**	**0.015**	**<0.001**	**<0.001**
	Hypoxia	51%	4%	18%	100%	**<0.001**	**<0.001**	**<0.001**	**<0.001**	**0.001**	**<0.001**	**<0.001**

Table S2. Comparison of reactive oxygen species (ROS) values of four different *Drosophila* strains in normoxia and hypoxia (analysis of variance with post hoc tests). In addition, comparison between normoxia and hypoxia ROS values for each strain (Student's *t*-test). ROS values are reported as mean ± standard deviation and *p*-values of pairwise comparisons are adjusted by means of Bonferroni correction

O_2 condition	Berlin-K	Canton-S	Oregon-R	*$Sod1^{n1}$*	*p*-value ANOVA	Berlin-K *vs.* Canton-S	Berlin-K *vs.* Oregon-R	Berlin-K *vs.* *$Sod1^{n1}$*	Canton-S *vs.* Oregon-R	Canton-S *vs.* *$Sod1^{n1}$*	Oregon-R *vs.* *$Sod1^{n1}$*
Normoxia, µmol/flies	0.047 ± 0.008	0.04 ± 0.002	0.042 ± 0.001	0.23 ± 0.017	**<0.001**	1.000	1.000	**<0.001**	1.000	**<0.001**	**<0.001**
Hypoxia, µmol/flies	0.16 ± 0.027	0.092 ± 0.02	0.138 ± 0.025	0.303 ± 0.07	**<0.001**	**0.017**	1.000	**<0.001**	0.264	**<0.001**	**<0.001**
Normoxia *vs.* hypoxia, *p*-value	**<0.001**	**0.002**	**0.015**	0.452							
Mean % increase after Hypoxic treatment	+243%	+129%	+232%	+34%							

Biology Open • Supplementary information
